# A Common Polymorphism near the ESR1 Gene Is Associated with Risk of Breast Cancer: Evidence from a Case-Control Study and a Meta-Analysis

**DOI:** 10.1371/journal.pone.0052445

**Published:** 2012-12-18

**Authors:** Hui Guo, Jie Ming, Chunping Liu, Zhi Li, Ning Zhang, Hongtao Cheng, Wei Wang, Wei Shi, Na Shen, Qunzi Zhao, Dapeng Li, Pengfei Yi, Longqiang Wang, Rui Wang, Yue Xin, Xiangwang Zhao, Xiu Nie, Tao Huang

**Affiliations:** 1 Department of Breast and Thyroid Surgery, Union Hospital, Tongji Medical College, Huazhong University of Science and Technology, Wuhan, People's Republic of China; 2 Department of Pathology, Union Hospital, Tongji Medical College, Huazhong University of Science and Technology, Wuhan, People's Republic of China; 3 Department of Breast Surgery, Hubei Cancer Hospital, Wuhan, People's Republic of China; Huazhong University of Science and Technology, China

## Abstract

**Background:**

Genome-wide association studies have reported that a polymorphism near the estrogen receptor gene (ESR1) (rs2046210) is associated with a risk of breast cancer, with the A allele conferring an increased risk. However, considering the controversial results from more recent replicated studies, we conducted a case-control study in an independent Chinese Han population and a meta-analysis to clarify the association of this polymorphism with breast cancer risk.

**Method and Findings:**

A hospital-based case-control study including 461 cases and 537 controls from a Chinese Han population was conducted initially, and this study showed that the rs2046210 A allele was significantly associated with breast cancer risk, with an OR of 1.32 (95% CI  = 1.10–1.59). Subsequently, a meta-analysis integrating the current study and previous publications with a total of 53,379 cases and 55,493 controls was performed to further confirm our findings. Similarly, a significant association between this polymorphism and breast cancer risk was also observed in the overall population especially among Asians, with ORs for per A allele of 1.14 (95% CI  = 1.10–1.18) in the overall population and 1.27 (95% CI  = 1.23–1.31) in the Asian population.

**Conclusion:**

Our results provide strong evidence to support that the common polymorphism near the ESR1 gene, rs2046210, is associated with an increased risk of breast cancer in Asian and European populations but not in Africans, although the biological mechanisms need to be further investigated.

## Introduction

According to global cancer statistics in 2008, breast cancer (BC) is the most common malignant tumor among women worldwide, accounting for 23% of new cancer cases and 14% of cancer deaths. In several developing countries, such as China, BC has surpassed cervical cancer and become the leading cause of cancer death among females [1]. Although the underlying mechanism of BC pathogenesis is still unclear, accumulating evidence shows that development of BC is a multifactorial complex process influenced by multiple genetic variants and environmental factors [2]. Given that only a few individuals receiving the same environmental exposure will develop BC, and that women with a family history of BC are at high risk for the cancer, it appears that genetic factors may play an important role in the etiology of BC. Previous studies have revealed that rare but high-penetrance mutations in BRCA1/2 and a few other inherited variants explain only up to 5% of overall BC incidence, whereas more common but low-penetrance susceptibility alleles may be responsible for a substantial fraction of BC [3]–[5].

Recent genome-wide association studies (GWAS) have demonstrated numerous low-penetrance susceptibility loci were significantly associated with BC [6]–[17]. Among them, a common single nucleotide polymorphism (SNP) in the vicinity of the ESR1 gene was highlighted for its potentially biological plausibility in the development of breast carcinogenesis. ESR1 gene encodes estrogen receptor α (ERα), which stimulates proliferation and differentiation of mammary epithelial tissue through combining with estrogen, an established risk factor for BC [18]. Because the biological roles of estrogen are achieved through a high-affinity combination with ERs, the genetic variants in ER genes have become the focus of molecular epidemiological studies on BC susceptibility [19], [20]. rs2046210, which is located 180 kb upstream of the transcription initiation site of the first coding exon of the ESR1 gene, was firstly reported by Zheng et al. [21], to be associated with an increased risk of BC. However, several subsequent replication studies could not reach consistent results; for example, Stacey et al. [22] failed to validate the association in Europeans, and similarly, Campa et al. [23] were also unable to replicate the findings in Asians. Potential explanations for the discrepancy could be the modest effect of this SNP and the diverse genetic backgrounds of the different ethnic groups. Additionally, due to the “winner's curse” phenomenon, the replication studies were likely to be underpowered and possibly failed if the sample size calculations were based on the overestimated effect sizes generated from the initial study [24]. Nevertheless, meta-analysis, a powerful tool that combines data to give the exponential increase in sample sizes, could resolve the discordances in genetic association studies [25]. Thus, in this study, we carried out a case-control study to validate the association of rs2046210 and BC risk in the Han Chinese population along with a meta-analysis that integrated the current study and previous publications about this polymorphism, to derive a more accurate estimation of the association between this polymorphism and BC risk.

## Materials and Methods

### Study population

A total of 461 incident cases and 537 controls were enrolled between June 2009 and December 2011 from Union Hospital of Huazhong University of Science and Technology, Wuhan, China. All the cases were histopathologically confirmed with primary BC and none of them had received neo-adjuvant treatment. The controls were randomly selected from a health check-up program at the same hospital during the same period. The inclusion criteria for controls were that they had to be cancer-free individuals who were frequently age-matched (±5 years) to the BC cases. All subjects were unrelated ethnic Han Chinese from Wuhan and its surrounding region in the central region of China. Written informed consent was signed by each participant, and 2-ml peripheral blood samples were collected on recruitment. According to the NCCN (National Comprehensive Cancer Network) guidelines for BC [26], the criteria for determining menopause include any of the following: (1) prior bilateral oophorectomy; (2) age ≥60 y; (3) age<60 y and amenorrheic for 12 or more months in the absence of chemotherapy, tamoxifen, toremifene, or ovarian suppression and FSH and estradiol in the postmenopausal range; (4) if taking tamoxifen or toremifene, and age <60 y, then FSH and plasma estradiol level in postmenopausal ranges. This study was approved by the institutional review boards of Union Hospital of Huazhong University of Science and Technology and Tongji Medical College of Huazhong University of Science and Technology.

### Genotyping

For each subject, genomic DNA was extracted from a 2-ml peripheral blood sample using the RelaxGene Blood System DP319-02 (Tiangen, Beijing, China) according to the manufacturer's instructions. The genotype of rs2046210 was carried out using the Taqman SNP Genotyping Assay (Applied Biosystems, Foster city, CA) on a 7900HT Fast Real-Time PCR System (Applied Biosystems, Foster city, CA). To assess the quality of genotyping, we conducted re-genotyping of a randomly selected 5% of the samples and obtained 100% concordance rate.

### Statistical Analysis

Differences in the distribution of demographic characteristics between the cases and controls were evaluated by using the *χ^2^* test and *t*-test. The Hardy-Weinberg equilibrium (HWE) for genotypes in the controls was assessed by the goodness-of-fit *χ^2^* test. An unconditional multivariate logistic regression model was used to estimate the associations between genotypes and BC risk by calculating the odds ratios (ORs) and 95% confidence intervals (CIs) after adjusting for age and menopausal status. To avoid the assumptions of genetic models, dominant, recessive and additive models for rs2046210 were also assessed. In addition, stratified analyses by menopausal status, estrogen receptor (ER) and progesterone receptor (PR) status were further carried out to evaluate the role of rs2046210 in BC risk. All statistical tests were two-sided and performed using the SPSS 12.0 computer program.

### Meta-analysis of rs2046210 in association with BC risk

To further investigate the association between rs2046210 and BC risk, we carried out a meta-analysis combining previous publications and the current study. We searched PubMed, EMBASE, ISI Web of Science databases and CNKI (China National Knowledge Infrastructure) for literature published in any language up to June 2012 using the keywords combinations of “rs2046210 or 6q25.1” and “breast cancer, breast carcinoma or breast neoplasm”. The references of retrieved articles and reviews were also checked for missing information. The literature that was included needed to meet the following criteria: (1) the study evaluating the association between rs2046210 and BC risk; (2) providing data for calculating genotypic ORs with corresponding 95% CI; (3) the genotypes in control conforming to Hardy-Weinberg equilibrium (*P*>0. 05). Reviews, simple commentaries and case reports were excluded. If the studies had overlapping subjects, the one with the largest samples was finally included.

For each study, the following data were extracted: first author, year of publication, geographic location, ethnicity of the study population, study design, study method, control source, sample size, and frequencies of genotypes in cases and controls. The ORs were calculated for the risk allele A versus the wild allele G. Genotype AG versus GG, AA versus GG, dominant, recessive and additive models were recalculated from parts of the included studies because some research did not provide sufficient data. The between-study heterogeneity was assessed by Cochran's *Q* test and *I^2^* statistics. Heterogeneity was considered significant at *P*<0.10 for the *Q* statistic [27]. The *I^2^* statistics was then used to evaluate heterogeneity quantitatively (*I^2^*<25%, low heterogeneity; *I^2^* = 25–75%, moderate heterogeneity; *I^2^*>75%, high heterogeneity) [28]. A fixed-effects model of the Mantel-Haenszel method was applied to pool data from studies if the heterogeneity was negligible based on a *P* value greater than 0.1 for *Q* statistics; otherwise, a random-effects model of the DerSimonian and Laird method was used [29]. To explore sources of heterogeneity across studies, a meta-regression model was employed [30]. The particular covariates for assessment of heterogeneity sources were: ethnicity (Asian, European and African), study design (GWA studies and replication), study method (case-control studies and nested case-control studies), sample size (≤2000 and >2000 subjects), source of control (population and hospital based controls). Stratified analysis was then conducted according to the potential sources of heterogeneity evaluated by meta-regression analysis. The subgroup meta-analyses stratified by ER and menopausal status were further performed. Additionally, sensitivity analysis was performed by omission of each study in turn to assess the influence of each study on the overall estimate [31]. Cumulative meta-analysis was performed by assortment of publication times [32]. Publication bias was assessed by a funnel plot and Eegger's test [33], [34]. All statistical analyses were carried out in STATA 10.0, and all *P* values were two-sided with a significance level at 0.05.

In order to ensure the rigor of this current meta-analysis, we designed and reported it according to the Preferred Reporting Items for Systematic Reviews and Meta-analyses (PRISMA) statement and the checklist is shown in Table S1 (http://www.prisma-statement.org).

## Results

### Results of case-control study

#### Population characteristics

The characteristics of the cases and controls were listed in Table 1. A total of 461 BC cases and 537 frequency-matched controls were enrolled in this study. Mean age was 48.41 (±9.85) for cases and 49.04 (±12.45) for controls, and there was no significant difference between two groups (*P* = 0.369). The percentage of premenopausal women was 54.7% among the BC cases compared with 46.6% among controls, and the *P* value for the distribution of menopause status between the cases and controls was 0.011.

**Table 1:** Characteristics of the study population.

Variables	Case (N = 461) No. (%)	Control (N = 537) No. (%)	*P* Value
Age (years)	48.41±9.85	49.04±12.45	0.369 a
Menopausal status			
Premenopausal	252(54.7)	250(46.6)	
Postmenopausal	209(45.3)	287(53.4)	0.011^b^
ER status			
ER Positive	266(59.5)		
ER Negative	181(40.5)		
PR status			
PR Positive	242(54.1)		
PR Negative	205(45.9)		

Abbreviations: ER, estrogen receptor; PR, progesterone receptor.

a *P* value was calculated by the *t* test.

b *P* value was calculated by the *χ^2^* test.

#### Association between SNP rs2046210 and BC risk

The genotype data of rs2046210 in the cases and controls are shown in Table 2. Genotypes in the controls were consistent with the Hardy-Weinberg equilibrium (*P*>0.05). A multivariate logistic regression model demonstrated that individuals carrying the A allele, GA or AA genotypes presented a significantly elevated risk of BC compared with those carrying the G allele or GG genotypes after adjusting for age and menopausal status (A versus G: OR = 1.32, 95% CI  = 1.10–1.59, *P* = 0.003; GA versus GG: OR = 1.34, 95% CI  = 1.02–1.76, *P* = 0.036; AA versus GG: OR  = 1.74, 95% CI  = 1.17–2.57, *P* = 0.006). Meanwhile, under dominant, recessive, and additive models, significant associations were also found for SNP rs2046210, with ORs equal to 1.42(95% CI = 1.10–1.84, *P* = 0.008), 1.49(95% CI  = 1.03–2.14, *P* = 0.034), and 1.32 (95% CI = 1.10–1.59, *P* = 0.003), respectively.

**Table 2:** Association between rs2046210 and breast cancer risk in a Han Chinese population.

	No. of cases	No. of controls			
	GG/GA/AA	GG/GA/AA	Genetic models	OR (95% CI)	*P* value
**All women**	164/222/75	236/240/61	A VS. G	1.32(1.10–1.59) a	0.003 a
			GA VS. GG	1.34(1.02–1.76) a	0.036 a
			AA VS. GG	1.74(1.17–2.57) a	0.006 a
			Dominant model	1.42(1.10–1.84) a	0.008 a
			Recessive model	1.49(1.03–2.14) a	0.034 a
			Additive model	1.32(1.10–1.59) a	0.003 a
**Menopausal status**					
Premenopausal	87/123/42	117/97/36	A VS. G	1.36(1.05–1.77) b	0.020 b
Postmenopausal	77/99/33	119/143/25	A VS. G	1.30(1.00–1.70) b	0.048 b
**ER status**					
ER Positive	93/135/38	236/240/61	A VS. G	1.27(1.03–1.58) a	0.029 a
ER Negative	68/76/37	236/240/61	A VS. G	1.38(1.08–1.77) a	0.009 a

Abbreviations: OR, odds ratio; 95%CI, 95% confidence interval.

a Data were calculated by multivariate logistic regression model after adjusting for age and menopausal status.

b Data were calculated by multivariate logistic regression model after adjusting for age.

We then stratified the data according to menopausal status and ER status. The results demonstrated that rs2046210 was associated with an elevated risk of BC in an allelic model among both pre- and post-menopausal individuals. The positive association of this SNP with BC risk was also found for both ER positive and ER negative women with adjusted ORs equal to 1.27(*P* = 0.029) and 1.38(*P* = 0.009) respectively.

### Result of meta-analyses

#### Characteristics of included studies

As shown in Figure S1, 23 potentially relevant publications were identified through PubMed, EMBASE, ISI Web of Science and CNKI initially, of which 17 publications were judged to preliminarily meet the inclusion criteria mentioned above. Seven articles were excluded because the cases largely overlapped with the samples of previous studies [35]–[41]. The multicenter research reported by Cai et al. [42] contained samples that duplicated those in the research conducted by Han et al [43]; therefore the corresponding study with less case number was excluded. Finally, 10 previous publications [21]–[23], [42]–[48] (Table 3) and the current study comprising 36 studies consisting of 53,379 cases and 55,493 controls were included in this meta-analysis based on our search strategy and eligibility criteria. Among them, the publication by Stacey et al. [22] provided only allelic OR value, and was thus only included in the pooled analysis for the allelic model of A VS. G. The study reported by Jiang et al. [47] did not provide the genotype of samples in detail, so we merely put it into the corresponding pooled analysis according to the data it provided.

**Table 3:** Characteristics of the studies included in the meta-analysis.

First Author	Published Year	Country	Ethnicity	Study Design	Study Method	Control Source	Case/Control
Zheng [21]	2009	China	Asian	GWAS and Replication	CC	PB	6472/3962
	2009	USA	European	Replication	CC	PB	1591/1466
Stacey [22]	2010	Iceland	European	Replication	CC	PB	2638/3506
	2010	USA	European	Replication	CC	PB	1753/1487
	2010	Spain	European	Replication	CC	PB	1009/1719
	2010	Netherlands	European	Replication	CC	PB	727/1830
	2010	Sweden	European	Replication	CC	PB	818/1750
	2010	Sweden	European	Replication	CC	PB	954/942
	2010	Nigeria	African	Replication	CC	PB	851/781
	2010	USA	African	Replication	CC	HB	300/153
	2010	Taiwan	Asian	Replication	CC	HB	1126/1118
Antoniou [48]	2011	Multinational	European	Replication	Nested CC	PB	5515/5302
	2011	Multinational	European	Replication	Nested CC	PB	3381/2807
Cai [42]	2011	China	Asian	Replication	CC	HB	1532/1583
	2011	China	Asian	Replication	CC	HB	1001/1013
	2011	China	Asian	Replication	CC	HB	407/421
	2011	Japan	Asian	Replication	CC	HB	644/644
	2011	Japan	Asian	Replication	Nested CC	PB	541/507
	2011	Japan	Asian	Replication	CC	HB	403/403
	2011	USA	European	Replication	CC	PB	1828/1438
	2011	USA	European	Replication	CC	PB	953/979
	2011	Multinational	European	Replication	Nested CC	PB	1145/1142
	2011	USA	African	Replication	Nested CC	PB	522/1046
	2011	USA	African	Replication	CC	PB	290/176
Jiang [47]	2011	China	Asian	Replication	CC	PB	493/510
Han [46]	2011	Korea	Asian	Replication	CC	PB	3251/3493
Stevens [44]	2011	Multinational	European	Replication	CC	PB and HB	2707/1385
Campa [23]	2011	USA	European	Replication	Nested CC	PB	603/817
	2011	Multinational	European	Replication	Nested CC	PB	2315/3217
	2011	USA	European	Replication	Nested CC	PB	2008/3710
	2011	Multinational	European	Replication	Nested CC	PB	666/668
	2011	Multinational	European	Replication	Nested CC	PB	1780/2168
	2011	Multinational	African	Replication	Nested CC	PB	402/426
	2011	Multinational	Asian	Replication	Nested CC	PB	524/537
Han [43]	2011	China	Asian	Replication	CC	PB	1768/1850
Guo	2012	China	Asian	Replication	CC	HB	461/537

Abbreviations: GWAS, genome-wide association studies; CC, case-control study; PB, population based; HB, hospital based.

#### Overall meta-analyses of rs2046210 in associated with BC risk

**Figure 1:**
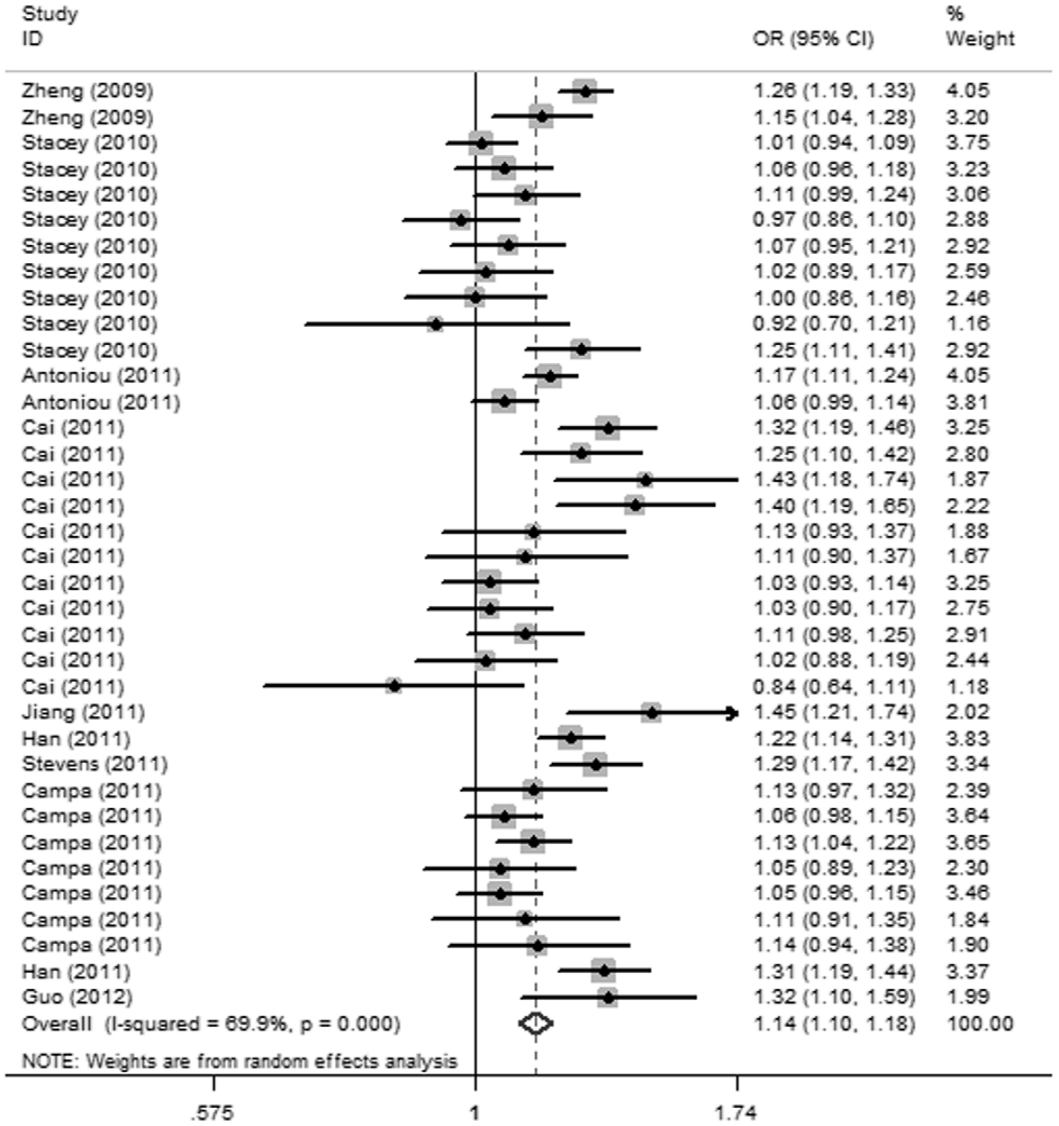
Forest plot of association between rs2046210 and BC risk under allelic model.

As shown in Table 4, the *P* values for heterogeneity were less than 0.1 in all genetic models, therefore, ORs were pooled under a random-effects model. In the allelic model, A allele conferred a pooled OR of 1.14 (95% CI  = 1.10–1.18, *P*<0.001) compared to G allele (Figure 1). Genotypic ORs of GA versus GG, AA versus GG, and a dominant model combined both crude and adjusted ORs because a study of Asians only provided adjusted ORs of the three models as mentioned previously [47], and the pooled ORs were 1.17 (95% CI = 1.11–1.24, *P*<0.001), 1.33 (95% CI  = 1.24–1.44, *P*<0.001) and 1.21 (95% CI = 1.14–1.28, *P*<0.001) respectively. Significant associations between rs2046210 and BC risk were also observed in the recessive model (OR  = 1.21, 95% CI = 1.15–1.28, *P*<0.001) and the additive model (OR = 1.15, 95% CI  = 1.11–1.20, *P*<0.001) in this meta-analysis.

**Table 4:** Meta-analysis of the rs2046210 in association with breast cancer risk under different genetic models.

	Genetic model	OR(95%CI)	*P*	*I^2^* (%)	*P*_ heterogeneity_	*P* for Egger's test
**Overall**						
Overall(n = 36)	A VS. G	1.14(1.10–1.18)	<0.001	69.9	<0.001	0.464
Overall (n = 26) a	AG VS. GG	1.17(1.11–1.24)	<0.001	59.1	<0.001	0.420
	AA VS. GG	1.33(1.24–1.44)	<0.001	57.9	<0.001	0.798
	Dominant model	1.21(1.14–1.28)	<0.001	66.1	<0.001	0.537
Overall(n = 25)	Recessive model	1.21(1.15–1.28)	<0.001	35.1	0.044	0.891
	Additive model	1.15(1.11–1.20)	<0.001	64.9	<0.001	0.495
**Ethnicity**						
** Asian**						
Asian(n = 13)	A VS. G	1.27(1.23–1.31)	<0.001	0.0	0.486	0.661
Asian(n = 12) a	AG VS. GG	1.30(1.24–1.37)	<0.001	11.6	0.331	0.773
	AA VS. GG	1.58(1.47–1.69)	<0.001	0.0	0.600	0.690
	Dominant model	1.36(1.30–1.42)	<0.001	14.3	0.305	0.931
Asian(n = 11)	Recessive model	1.36(1.28–1.45)	<0.001	0.0	0.546	0.539
	Additive model	1.26(1.22–1.31)	<0.001	0.0	0.517	0.966
** European**						
European(n = 18)	A VS. G	1.09(1.07–1.12)	<0.001	47.8	0.013	0.299
European (n = 11)	AG VS. GG	1.11(1.07–1.16)	<0.001	37.3	0.101	0.082
	AA VS. GG	1.20(1.14–1.28)	<0.001	0.0	0.670	0.331
	Dominant model	1.13(1.09–1.18)	<0.001	34.9	0.120	0.084
	Recessive model	1.14(1.08–1.20)	<0.001	0.0	0.844	0.885
	Additive model	1.10(1.07–1.13)	<0.001	11.4	0.335	0.141
** African**						
African(n = 5)	A VS. G	1.01(0.92–1.09)	0.977	0.0	0.553	0.287
African(n = 3)	AG VS. GG	1.03(0.82–1.28)	0.826	66.5	0.051	0.596
	AA VS. GG	1.03(0.82–1.30)	0.799	47.1	0.151	0.556
	Dominant model	1.03(0.84–1.27)	0.785	65.3	0.056	0.562
	Recessive model	1.02(0.87–1.19)	0.837	0.0	0.888	0.221
	Additive model	1.02(0.91–1.13)	0.782	18.6	0.293	0.549
**ER status** b						
ER positive(n = 7)	A VS. G	1.12(1.04–1.20)	0.002	77.2	<0.001	0.942
ER negative(n = 8)	A VS. G	1.23(1.14–1.32)	<0.001	73.6	<0.001	0.424
**Menopausal status** c						
Premenopausal(n = 5)	A VS. G	1.18(1.13–1.24)	<0.001	45.7	0.118	0.945
Postmenopausal(n = 5)	A VS. G	1.22(1.10–1.36)	<0.001	79.8	0.001	0.907

a Included a study that only offered the adjusted ORs for AG vs. GG, AA vs. GG and dominant model, both crude and adjusted ORs were combined.

b Compared to cases with ER positive cancer, crude OR(95% CI) for ER negative cancer was 1.11(1.06–1.15) for the model of A VS. G (*P* = 8.27×10^−7^).

c Compared to premenopausal cases, crude OR(95% CI) for postmenopausal cases was 0.99(0.95–1.04) for the model of A VS. G (*P* = 0.706).

#### Meta-regression analyses and stratified analyses

**Figure 2:**
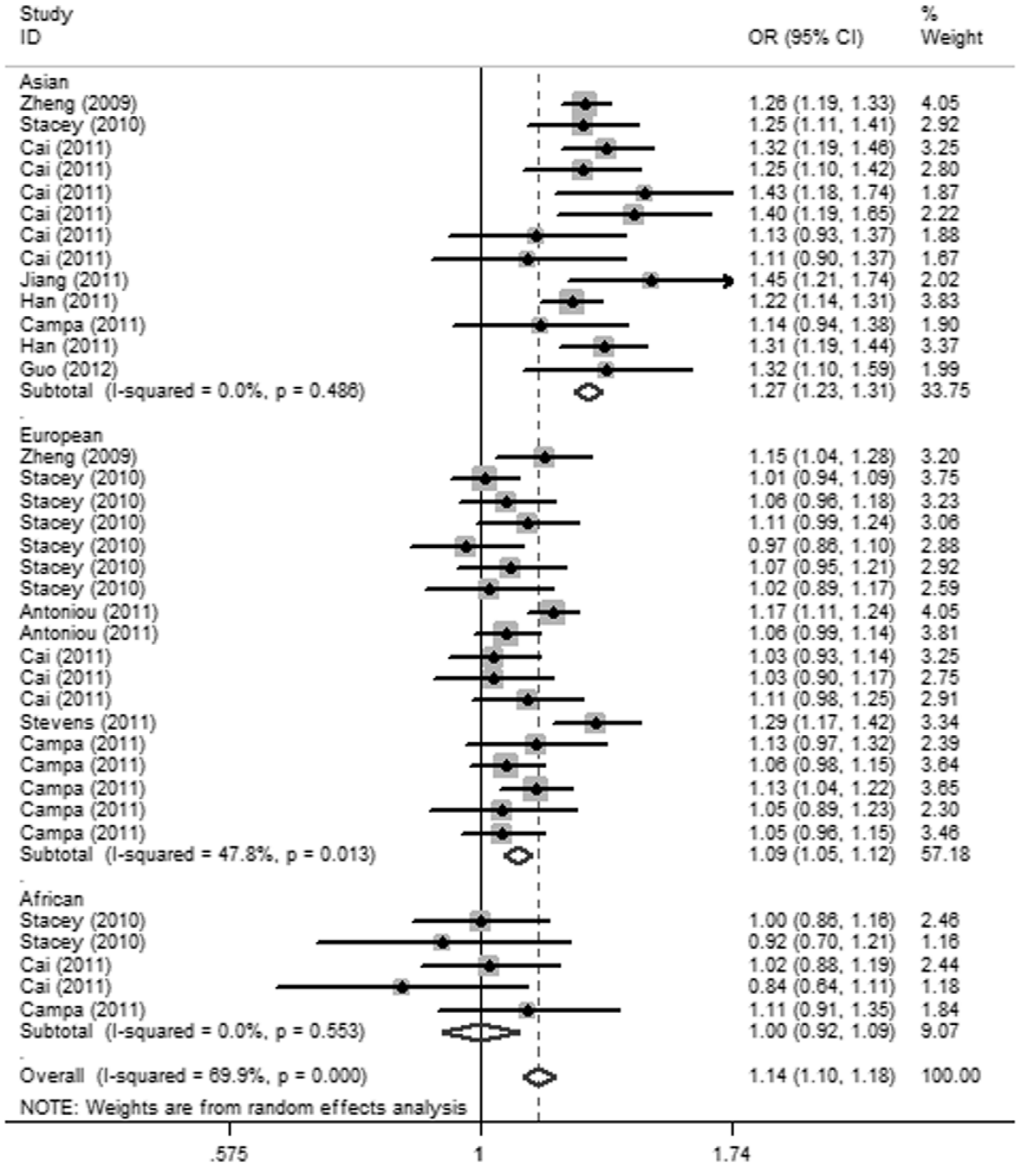
Forest plot of association between rs2046210 and BC risk in different ethnicities under allelic model.

To investigate the potential sources of between-study heterogeneity under allelic model of A VS. G, meta-regression analyses were performed. A empty regression was initially run to estimate the baseline value for *tau* ^2^ (*tau* ^2^  = 0.0073), and then we conducted a series of univariate model by adding single covariates including ethnicity of population, study design, study method, sample size, and source of control. In the univariate analyses, we found that the *tau* ^2^ value reduced to 0.0014 (adjusted *R* ^2^ = 81.04%) in the model of ethnicity, suggesting that ethnicity could explain 81.04% of the heterogeneity across studies in this allelic model. Then the stratified analyses by ethnicity were further carried out (Table 4). In Asian and European populations, the polymorphism in all genetic models presented a significantly increased risk of BC; however, there was no obvious association between the SNP and BC risk in the African population in any genetic model (Figure 2). It demonstrated that the A variant played disparate roles in different ethnic populations. We found that the moderate heterogeneity still exited in the Europeans under allelic model (*I* ^2^ = 47.8%, *P* = 0.013), therefore, the further meta-regression was carried out and it revealed that the source of control could explain 53.61% of heterogeneity. After excluding the multicenter research reported by Steven et al [44] that combined hospital- and population-based controls together, the heterogeneity was reduced apparently (*I* ^2^ = 21.6%, *P* = 0.202).When we subsequently stratified the data by ER and menopausal status, the between-study heterogeneity was obvious, but it was reduced notably after further stratifying by ethnicity. The pooled ORs of A VS. G were 1.23(95% CI = 1.14–1.32, *P*<0.001) in the ER negative population and 1.12(95% CI  = 1.04–1.20, *P* = 0.002) in the ER positive population. After comparing to cases with ER positive BC, the OR (95%CI) for ER negative BC was 1.11(95% CI = 1.06–1.15, *P* = 8.27×10^−7^) for allelic model of A VS.G, which indicated that the association was stronger for ER negative BC than ER positive BC. Meanwhile, the positive association of this SNP with BC risk was also found in both pre- and post-menopausal women (OR = 1.18, 95% CI = 1.13–1.24, *P*<0.001 and OR = 1.22, 95% CI = 1.10–1.36, *P*<0.001), however, no stronger association was found in post-menopausal cases by comparison with pre-menopausal counterparts (*P* = 0.706).

#### Sensitivity analyses and cumulative meta-analyses

Since significant heterogeneity across studies existed in all genetic models of overall population and in allelic model of European population, we carried out sensitivity analyses to evaluate the effect of each study on the pooled estimate under a random-effects model by removing each study sequentially. As shown in Table 5 and Table S2, the pooled ORs were similar before and after deletion of each study. We also achieved similar results in other genetic models and no single study changed the OR values markedly, therefore, the current results are stable and credible.

**Figure 3:**
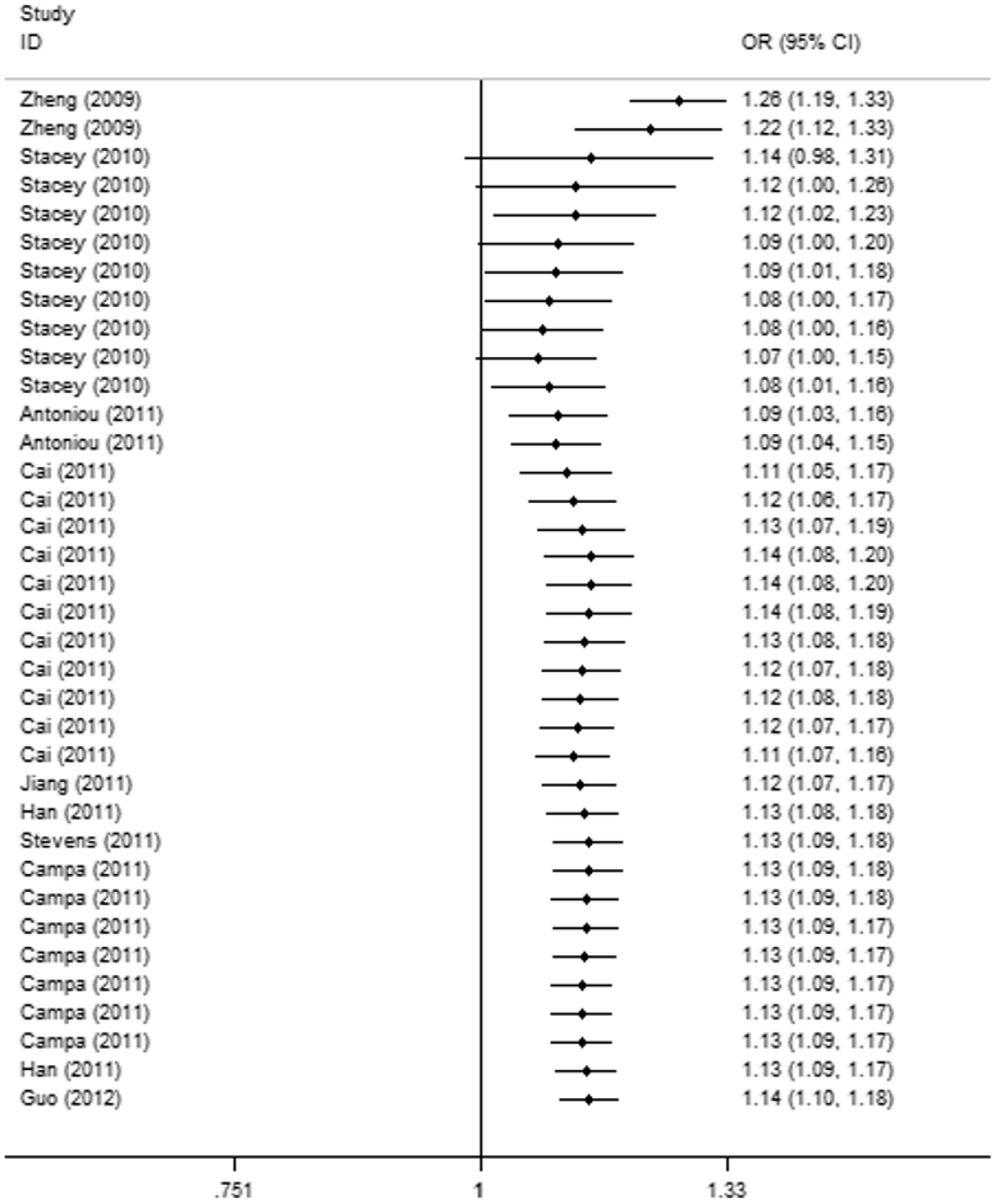
Cumulative meta-analysis of association between rs2046210 and BC risk under allelic model.

Cumulative meta-analyses were carried out in all genetic models via an assortment of studies in chronologic order. As shown in Figure 3, the 95% CIs for the pooled ORs became increasingly narrower with each accumulation of more studies in all models, indicating that the precision of the estimation was progressively boosted by continually adding more samples.

**Table 5:** Sensitivity analysis of allelic model.

Study omitted	OR(95%CI)	*P*_ heterogeneity_	*I^2^* (%)
Zheng 2009 China [21]	1.13(1.09–1.17)	<0.001	67.0
Zheng 2009 USA	1.14(1.10–1.18)	<0.001	70.7
Stacey 2010 Iceland [22]	1.14(1.10–1.18)	<0.001	67.6
Stacey 2010 USA1	1.14(1.10–1.18)	<0.001	70.2
Stacey 2010 Spain	1.14(1.10–1.18)	<0.001	70.7
Stacey 2010 Netherlands	1.14(1.10–1.18)	<0.001	68.9
Stacey 2010 Sweden1	1.14(1.10–1.18)	<0.001	70.5
Stacey 2010 Sweden2	1.14(1.10–1.18)	<0.001	70.1
Stacey 2010 Nigeria	1.14(1.10–1.18)	<0.001	69.9
Stacey 2010 USA2	1.14(1.10–1.18)	<0.001	70.1
Stacey 2010 China	1.13(1.09–1.17)	<0.001	70.2
Antoniou 2011 CIMBA1 [48]	1.13(1.09–1.18)	<0.001	70.5
Antoniou 2011 CIMBA2	1.14(1.10–1.18)	<0.001	69.5
Cai 2011 China1 [42]	1.13(1.09–1.17)	<0.001	68.6
Cai 2011 China2	1.13(1.09–1.17)	<0.001	70.2
Cai 2011 China3	1.13(1.09–1.17)	<0.001	69.4
Cai 2011 Japan1	1.13(1.09–1.17)	<0.001	69.2
Cai 2011 Japan2	1.14(1.10–1.18)	<0.001	70.7
Cai 2011 Japan3	1.14(1.10–1.18)	<0.001	70.7
Cai 2011 CBCS	1.14(1.10–1.18)	<0.001	69.7
Cai 2011 LIBCSP	1.14(1.10–1.18)	<0.001	70.1
Cai 2011 CGEMS	1.14(1.10–1.18)	<0.001	70.7
Cai 2011 SCCS	1.14(1.10–1.18)	<0.001	70.2
Cai 2011 NBHS	1.14(1.10–1.18)	<0.001	69.5
Jiang 2011 China [47]	1.13(1.09–1.17)	<0.001	69.0
Han 2011 Korea [46]	1.13(1.09–1.17)	<0.001	69.8
Stevens 2011 TNBCC [44]	1.13(1.09–1.17)	<0.001	69.1
Campa 2011 CPS2 [23]	1.14(1.10–1.18)	<0.001	70.7
Campa 2011 EPIC	1.14(1.10–1.18)	<0.001	69.8
Campa 2011 NHS	1.14(1.10–1.18)	<0.001	70.7
Campa 2011 WHS	1.14(1.10–1.18)	<0.001	70.5
Campa 2011 MEC+PLCO1	1.14(1.10–1.18)	<0.001	69.8
Campa 2011 MEC+PLCO2	1.14(1.10–1.18)	<0.001	70.7
Campa 2011 MEC+PLCO3	1.14(1.10–1.18)	<0.001	70.7
Han 2011 China [43]	1.13(1.09–1.17)	<0.001	68.5
Guo 2012 China	1.13(1.09–1.17)	<0.001	70.1

#### Publication bias

A funnel plot (Figure S2) and Egger's test (all P values for Egger's test >0.05) reflected that there was no evidence of publication bias in any of the genetic models.

## Discussion

This study demonstrated a significant association between rs2046210 and an increased risk of BC in a Han Chinese population. The subsequent meta-analysis based on 36 studies consisting of 53,379 cases and 55,493 controls also confirmed the strong association under all genetic models in an overall population. To the best of our knowledge, this is the first meta-analysis seeking to clarify the association between this polymorphism and BC risk, and the sensitivity and cumulative analyses confirmed that the positive finding was stable and the precision of estimation was progressively boosted as more studies were involved. These results clearly revealed the role of this polymorphism, which is near the ESR1 gene, in BC susceptibility.

In the overall meta-analyses, all genetic models presented significant heterogeneity. However, the heterogeneity had been mostly explained by the ethnicity of study population according to the result of meta-regression analyses. After being stratified by ethnicity, it demonstrated that this polymorphism had a significant association with BC risk in Asians and only a weaker and unstable association in Europeans. Meanwhile it could not be validated in Africans. The strength of the association with rs2046210 varies greatly across ethnic groups. One probable reason is the considerable differences in genetic architecture across ethnic SNPs. Another plausible hypothesis suggests that rs2046210 is only a marker SNP of causative variants and resides in different linkage disequilibrium (LD) patterns among the three ethnic populations.

Intriguingly, in further analysis, we found that this association was more significant in ER negative than in ER positive BC. Two recent interesting studies [45], [48] indicated that this polymorphism was associated with an increased risk of BC with BRCA1 mutation carriers, but not associated in BRCA2 mutation patients. Remarkably, accumulating evidence showed that the large majority of BRCA1 mutation carriers presented with ER negative tumors [49], which could partly explain why ER negative cases were accompanied by a stronger association. Additionally, recent studies in mice have revealed that the mammary stem cell compartment could be regulated by estrogen and progesterone through a paracrine signaling mechanism from ER positive cells to ER negative cells [50], [51]. Thus, polymorphisms near the ESR1 locus could affect the occurrence and development of ER negative tumors through the paracrine pathway. In the stratified meta-analysis, we also found that rs2046210 was significantly associated with BC risk in both premenopausal and postmenopausal women for allelic model, which was kept in line with the result of our case-control study. However, there was no evidence showing that the association was stronger in post- than pre-menopausal women.

Considering the relative vicinity of rs2046210 to the ESR1 gene, it was speculated that the SNP itself or causal variants in LD with it might alter ESR1 gene expression, thus affecting the susceptibility to BC. However, the functional genomic analyses and in vitro functional experiments conducted by Cai et. al [42] provided no support for the potential involvement of this polymorphism in the regulation of ESR1. Although dozens of SNPs have been reported in high LD with this polymorphism, functional evaluations on them and their related genes were still warranted. Herein, we conjectured that this SNP might communicate with the ESR1 gene via a long-range chromatin loop. Nevertheless, it was just a postulation and needed to be confirmed by further longitudinal studies.

In conclusion, our case-control study and the subsequent meta-analysis effectively corroborated the impact of rs2046210 near the ESR1 gene on BC risk, and showed that the polymorphism had a larger effect on Asians than on Europeans or Africans. However, the function of this SNP is still unclear; future fine-mapping of the BC susceptibility loci tagged by rs2046210 is warranted and the underlying biological mechanism of this polymorphism still needs further investigation.

## Supporting Information

Figure S1 Flow diagram of the study selection procedure.
(TIF)

Figure S2 Funnel plot for publication bias test.
(TIF)

Table S1 PRISMA Checklist.
(DOC)

Table S2 Sensitivity analysis of allelic model for Europeans.
(DOC)
