# Supplementary figures and images for: A Common Polymorphism near the ESR1 Gene Is Associated with Risk of Breast Cancer: Evidence from a Case-Control Study and a Meta-Analysis
Source: PLoS One. 2012 Dec 18;7(12):e52445. doi: 10.1371/journal.pone.0052445 (PMC3525547; doi:10.1371/journal.pone.0052445)

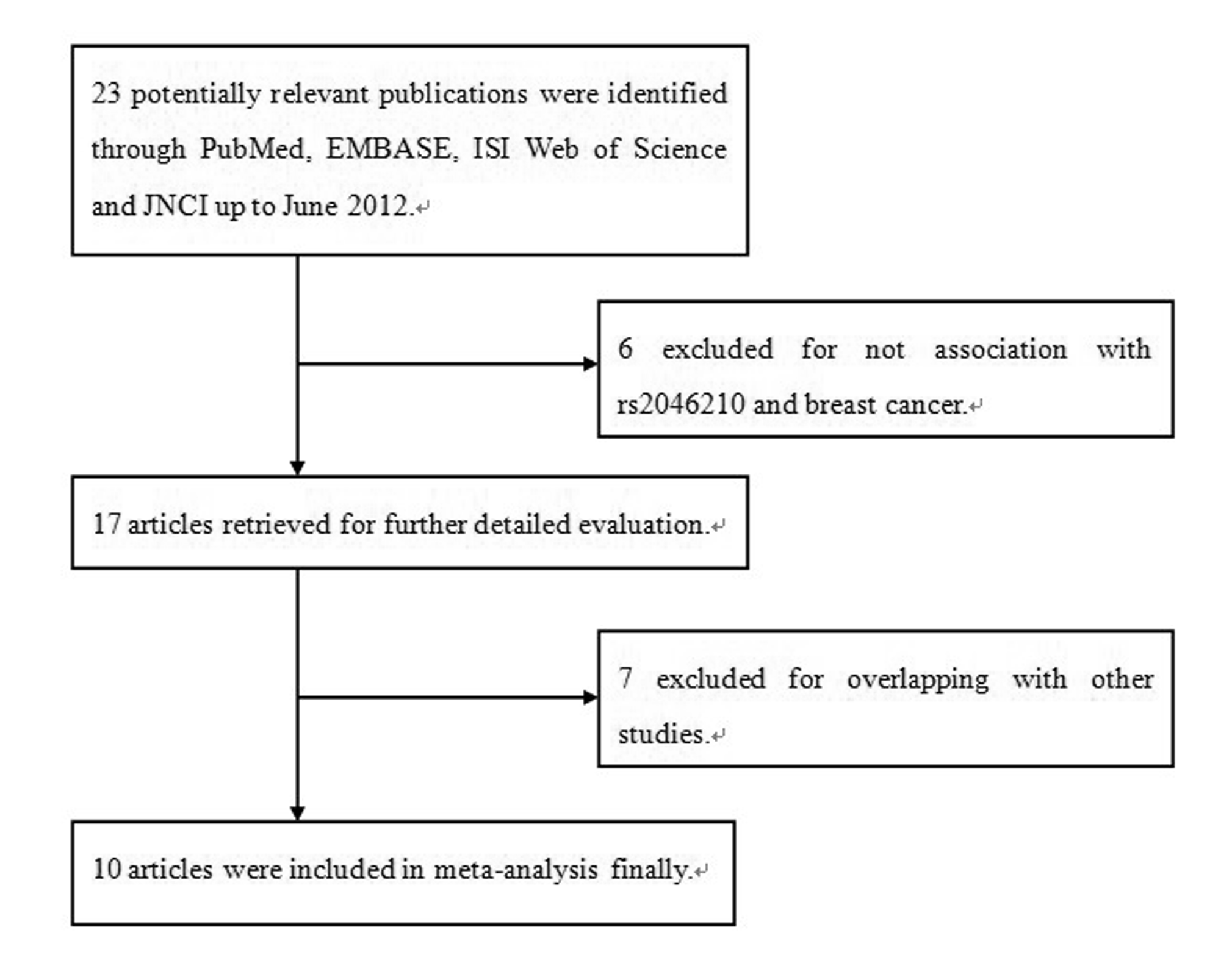

Supplement: Figure S1 — Flow diagram of the study selection procedure. (TIF) [file pone.0052445.s001.tif]

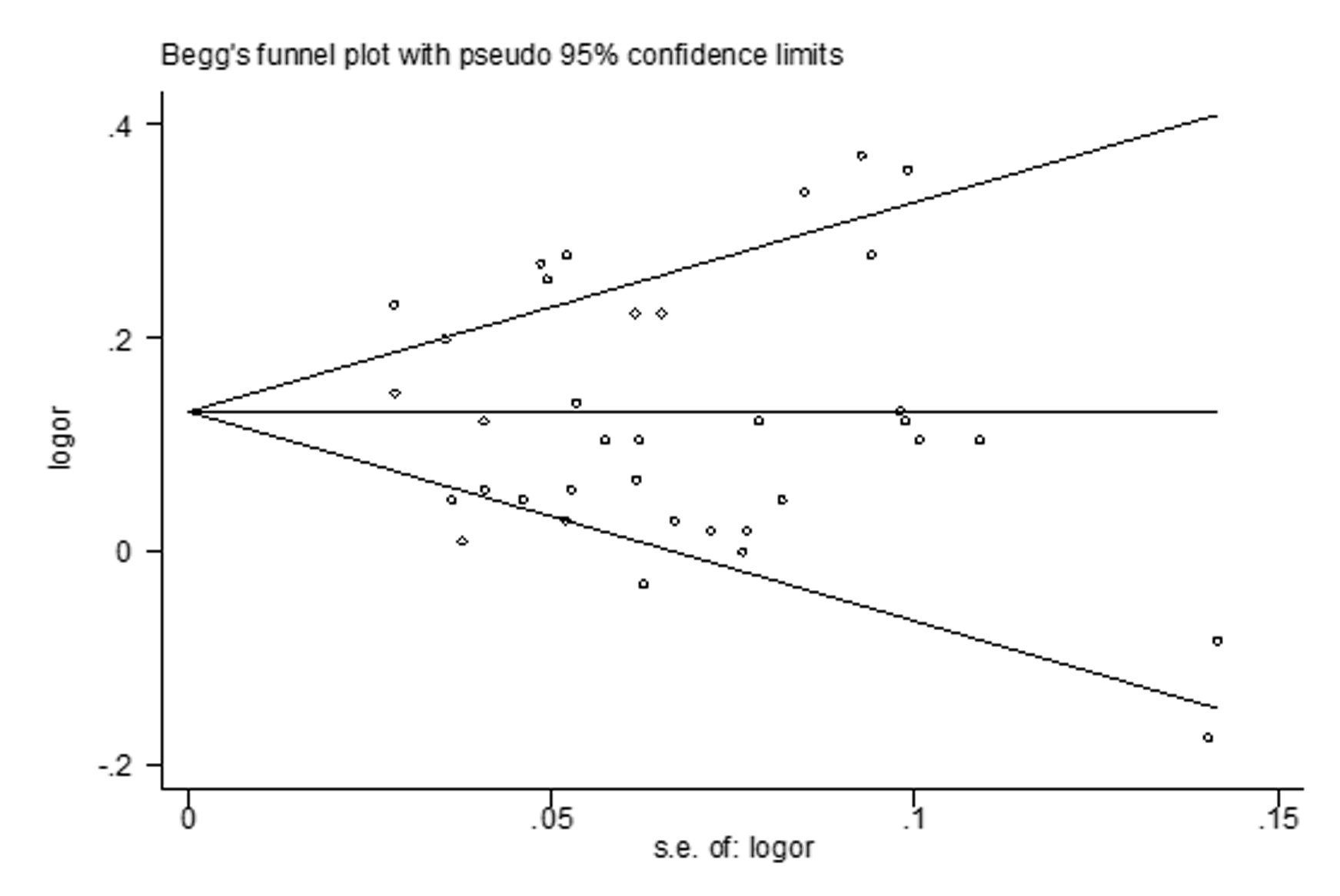

Supplement: Figure S2 — Funnel plot for publication bias test. (TIF) [file pone.0052445.s002.tif]
