# Supplementary material for: A Common Polymorphism near the ESR1 Gene Is Associated with Risk of Breast Cancer: Evidence from a Case-Control Study and a Meta-Analysis
Source: PLoS One. 2012 Dec 18;7(12):e52445. doi: 10.1371/journal.pone.0052445 (PMC3525547; doi:10.1371/journal.pone.0052445)
Supplement: Table S2 — Sensitivity analysis of allelic model for Europeans. (DOC) [file pone.0052445.s004.doc]

**Table S2. Sensitivity analysis of allelic model for Europeans.**

| **Study omitted** | **OR(95%CI)** | ***P* for heterogeneity** | ***I^2^* (%)** |
| --- | --- | --- | --- |
| Zheng 2009 USA | 1.08（1.05-1.12） | 0.011 | 49.3 |
| Stacey 2010 Iceland | 1.09（1.06-1.13） | 0.032 | 42.8 |
| Stacey 2010 USA1 | 1.09（1.05-1.12） | 0.009 | 50.4 |
| Stacey 2010 Spain | 1.08（1.05-1.12） | 0.009 | 50.7 |
| Stacey 2010 Netherlands | 1.09（1.06-1.13） | 0.024 | 44.7 |
| Stacey 2010 Sweden1 | 1.09（1.05-1.12） | 0.009 | 50.7 |
| Stacey 2010 Sweden2 | 1.09（1.05-1.12） | 0.011 | 49.5 |
| Antoniou 2011 CIMBA1 | 1.08（1.04-1.11） | 0.063 | 37.0 |
| Antoniou 2011 CIMBA2 | 1.09（1.05-1.13） | 0.010 | 49.8 |
| Cai 2011 CBCS | 1.09（1.05-1.13） | 0.012 | 48.9 |
| Cai 2011 LIBCSP | 1.09（1.05-1.13） | 0.011 | 49.7 |
| Cai 2011 CGEMS | 1.08（1.05-1.11） | 0.009 | 50.8 |
| Stevens 2011 TNBCC | 1.08（1.05-1.11） | 0.202 | 21.6 |
| Campa 2011 CPS2 | 1.08（1.05-1.12） | 0.009 | 50.6 |
| Campa 2011 EPIC | 1.09（1.05-1.13） | 0.010 | 50.1 |
| Campa 2011 NHS | 1.08（1.05-1.12） | 0.011 | 49.6 |
| Campa 2011 WHS | 1.09（1.05-1.12） | 0.009 | 50.5 |
| Campa 2011 MEC+PLCO1 | 1.09（1.05-1.13） | 0.011 | 49.7 |
